# Audit of cancer pain management practices at the adult oncology center, University of Gondar Comprehensive Specialized Hospital, Northwest Ethiopia

**DOI:** 10.3389/fonc.2025.1542227

**Published:** 2025-05-02

**Authors:** Temesgen Birlie Asmare, Hailu Yimer Tawuye, Biresaw Ayen Tegegne, Negesse Zurabachew Gobezie, Habtie Bantider Wubet, Gezahagn Demisu Gedefaw, Molla Amsalu Tadesse, Biruk Adie Admass

**Affiliations:** ^1^ Department of Anesthesia, School of Medicine, College of Health Science, Debre Tabor University, Debre Tabor, Ethiopia; ^2^ Department of Anesthesia, School of Medicine, College of Medicine and Health Sciences, University of Gondar, Gondar, Ethiopia; ^3^ Department of Neonatal Health Nursing, School of Nursing, College of Medicine and Health Science, and Specialized Hospital, University of Gondar, Gondar, Ethiopia; ^4^ Department of Anesthesia, Asrat Woldeyes Health Science Campus, Debre Berhan University, Debre Berhan, Ethiopia

**Keywords:** oncology, cancer, pain, pain management, adult

## Abstract

**Introduction:**

Cancer is one of the leading causes of death globally, with an estimated 19.3 million new cancer cases and nearly 10.0 million cancer deaths occurring in 2020. Pain is common among patients with cancer, particularly in the advanced stages of the disease, where the prevalence is estimated to exceed 70%. In our setting, the prevalence of cancer pain is high (59.9%), which makes the study of cancer pain management essential in order to identify specific gaps in current practices. This research aimed to enhance the quality of pain management and to improve patient care and safety in accordance with the European Society of Medical Oncology (ESMO) cancer pain management guidelines.

**Method:**

A hospital-based cross-sectional study was conducted from July 2 to August 1, 2024. All consecutive adult patients in the oncology ward during this period were included. Data were collected according to the ESMO guidelines through direct observations, chart reviews, and interviews. The data were then entered into EpiData version 4.6 and exported to Stata version 17 for analysis.

**Result:**

The study included 171 patients, of whom 96 (56.14%) were women. The overall compliance rate for oncologic pain management according with the ESMO guidelines was 55.46%. The ages of the participants ranged from 23 to 85 years, with a mean age of 51.2 years. The pain severity and the treatment outcomes were assessed regularly and consistently using the Numeric Rating Scale (NRS) for all 171 patients (100%). However, the subcutaneous route was considered as the first choice in only 2 (1.16%) patients who were unable to receive opioids via the oral route.

**Conclusion and recommendations:**

In this study, the overall compliance with the adult oncologic pain management guidelines was found to be suboptimal. It is recommended to establish a regular training program for healthcare professionals focusing on oncologic pain management.

## 1 Introduction

Cancer is one of the leading causes of death globally, but its burden is not evenly distributed (1). Worldwide, an estimated 19.3 million new cancer cases and nearly 10.0 million cancer deaths occurred in 2020. Female breast cancer has surpassed lung cancer as the most commonly diagnosed cancer, with approximately 2.3 million new cases (11.7%) (2).

Pain is common among patients with cancer, particularly in the advanced stages of the disease, where its prevalence is estimated to exceed 70% (3–5). A comprehensive systematic review indicates that the pain prevalence ranges from 33% in patients who have undergone curative treatment to 59% in those receiving anticancer therapy, increasing to 64% in patients with metastatic, advanced, or terminal disease (6). The consequences of undertreated cancer pain are both physical and psychological, resulting in suffering and a diminished quality of life. Physical effects may include insomnia, sleep disturbances, anorexia, decreased cognition, various forms of incapacity, and profound fatigue (7). Unrelieved pain can cause patients to withdraw from social and familial interactions, leading to feelings of isolation and psychological distress. Furthermore, persistent pain can result in existential and spiritual suffering, which may hinder patients’ coping abilities (7, 8).

Cancer pain management is a critical aspect of oncology care that significantly impacts patients’ quality of life. The European Society of Medical Oncology (ESMO) has established comprehensive guidelines to address this issue, emphasizing a multifaceted approach to pain management (9). A thorough pain assessment involves evaluation of the intensity, nature (acute or chronic), and causes of pain and one that supports a multidisciplinary strategy for effective pain management (10, 11). The ESMO recommendations advocate for a stepwise approach to pharmacological management, which largely aligns with the World Health Organization (WHO) analgesic ladder. In addition to pharmacological strategies, the ESMO also recommends integrating non-pharmacological approaches into cancer pain management. These approaches include radiotherapy, interventional techniques such as nerve blocks and spinal cord stimulation for patients with refractory pain, and psychological support (9, 12, 13).

In general, cancer-related pain can significantly affect patients’ quality of life, adherence to therapy, and satisfaction with their care (14). In our setup at the University of Gondar Comprehensive and Specialized Hospital (UoGCSH), the prevalence of cancer pain among adult cancer patients was 59.9% (15). Therefore, given the high prevalence of cancer pain in our setting, studying cancer pain management helps identify specific gaps in practice and enhances the quality of pain management. This ultimately leads to improved patient care and safety following the ESMO guidelines for cancer pain management.

## 2 Methods and materials

### 2.1 Study design, period, and area

A hospital-based cross-sectional study was conducted from July 2 to August 1, 2024, at the University of Gondar Comprehensive Specialized Hospital (UoGCSH) in Gondar, Ethiopia. UoGCSH has an isolated adult oncology ward with 32 beds and an average monthly admission rate of 176 patients. This ward is staffed by three medical oncologists, two internal medicine (hematology) residents, three general practitioners (GPs), two interns who rotate every 2 weeks, and 12 nurses. The most commonly admitted cases in this study include breast cancer, cholangiocarcinoma, cancer of unknown primary origin, cervical cancer, ovarian cancer, colorectal cancer, and Hodgkin’s lymphoma. Less common admissions include esophageal cancer, squamous cell carcinoma, and colon cancer.

### 2.2 Population

#### 2.2.1 Source population and study population

All patients admitted to the adult oncology ward constituted the source population. The study population included those who were admitted during the study period and met the inclusion criteria.

### 2.3 Inclusion and exclusion criteria

Patients aged over 18 years who were admitted to the adult oncology ward during the study period were included. However, those who underwent major surgery within the previous 2 weeks; those unable to communicate their pain due to cognitive impairment, delirium, or altered mental status; and those with pain-causing coexisting conditions (such as arthritis, diabetes, and fibromyalgia) were excluded.

### 2.4 Sample size and sampling technique

During the study period, 171 patients were admitted to the adult oncology ward and met the inclusion criteria. All of these patients were included in the study sample. Patients were recruited through consecutive sampling.

### 2.5 Variables of the study

#### 2.5.1 Compliance

Oncologic pain management is deemed compliant when it is provided according to the protocol. Any practices that deviate from the protocol are considered non-compliant.

#### 2.5.2 Compliance rate

This refers to the ratio of the number of patients who received the standard oncologic pain management care to the overall number of patients who were screened for that specific care.

### 2.6 Data collection instrument

The ESMO has developed standards for managing oncologic pain. Data were collected using the ESMO cancer pain management guidelines, which were transformed into a questionnaire format consisting of 14 questions with three response options: “Yes,” “No,” and “Not applicable.” The expected compliance rate for all oncologic pain management guidelines is 100% (**Table 1**). After converting the recommendations in the ESMO guidelines into question forms, the authors collaborated with language experts at the university to translate them into Amharic, the local language. The translations were then collated and retranslated into English.

**Table 1:** European Society of Medical Oncology (ESMO) guidelines on adult oncologic pain management.

S. no.	Variable	Target (%)	Evidence	Data source
1	The intensity of pain and the treatment outcomes should be assessed regularly and consistently using the VAS or NRS.	100	ESMO, 2018	Observation and chart review
2	The onset of pain should be prevented by employing around-the-clock (ATC) administration, taking into account the half-life, bioavailability, and duration of action of different drugs.	100	ESMO, 2018	Observation and chart review
3	Functional impairment should be assessed for moderate to severe pain.		ESMO, 2018	Observation
4	Patients should be informed about pain and pain management and should be encouraged to take an active role in their pain management.	100	ESMO, 2018	Interview and observation
5	Analgesics for chronic pain should be prescribed regularly and not on an “as required” schedule.	100	ESMO, 2018	Interview and chart review
6	The oral route of administration of analgesic drugs should be advocated as the first choice.	100	ESMO, 2018	Observation and chart review
7	Combination of step 1 analgesics [e.g., paracetamol or non-steroidal anti-inflammatory drugs (NSAIDs)] for mild pain with step 2 (weak opioids) for moderate pain or step 3 analgesics (strong opioids) for severe pain should be utilized.	100	ESMO, 2018	Observation and chart review
8	The opioid of first choice for moderate to severe cancer pain is oral morphine.	100	ESMO, 2018	Observation and chart review
9	A different opioid should be considered in the absence of adequate analgesia (despite opioid dose escalation) or the presence of unacceptable opioid side effects.	100	ESMO, 2018	Observation and chart review
10	The s.c. route is simple and effective for the administration of morphine. It should be the first-choice route for patients unable to receive opioids orally.	100	ESMO, 2018	Observation and chart review
11	Laxatives must be routinely prescribed for both the prophylaxis and the management of opioid-induced constipation.	100	ESMO, 2018	Chart review
12	The side effects of opioid medications should be recorded.	100	ESMO, 2018	Chart review
13	Cancer-related neuropathic pain can be treated using opioid combination therapies and carefully dosed adjuvants when opioids alone provide insufficient pain relief.	100	ESMO, 2018	Observation and chart review
14	Patients with neuropathic pain should be given either a TCA or an anticonvulsant and be monitored for side effects.	100	ESMO, 2018	Observation and chart review

VAS, Visual Analogue Scale; NRS, Numeric Rating Scale; TCA, tricyclic antidepressant.

### 2.7 Data collection procedure

A pretest was conducted 2 weeks before the actual data collection, which involved 5% (8) of the patients—they were excluded from the final analysis—at the University of Gondar Comprehensive Specialized Hospital. This sample size was based on the previous monthly admission rate in the adult oncology ward, which was 176. Data were collected using the ESMO guidelines through direct observations with a checklist, interviews, and chart reviews by two trained anesthesiology student interns. Patient-specific identifiers were not included to maintain confidentiality. Most of the medical records were assessed through a chart review and observations. Moreover, the patient interview was used for questions such as obtaining information from patients about pain and pain management and encouraging patients to take an active role in their pain management.

### 2.8 Data processing and analysis

The collected data were checked for completeness, accuracy, and clarity. Subsequently, the data were entered, coded, and cleaned using EpiData version 4.6. Descriptive analysis was conducted using Stata version 17. The results are presented in graphs, frequencies, and percentages.

## 3 Results

### 3.1 Socio-demographic clinical characteristics of the patients

This study included 171 patients, of whom 96 (56.14%) were women. The majority of the participants, 90 (52.63%), were aged between 41 and 65 years, with a mean age of 51.2 years (SD = 15.4) (**Table 2**). Among the participants, 35 (20.47%), 20 (11.70%), and 19 (9.94%) were diagnosed with breast cancer, cancer of unknown primary origin, and ovarian cancer, respectively (**Table 2**). More than two-thirds of the patients, 68 (39.76%), were diagnosed with stage IV cancer (**Table 2**).

**Table 2:** Socio-demographic and clinical characteristics of adult oncologic patients at the University of Gondar Comprehensive and Specialized Hospital, 2024 (*N* = 171).

Variable	Category	Percentage (*n*)
Age (years)	18–40	26.32 (45)
	41–65	52.63 (90)
	>65	21.05 (36)
	Mean (SD)	51.2 (15.4)
Sex	Women	56.14 (96)
	Men	43.86 (75)
Diagnosis	Acute myeloid lymphoma	7.02 (12)
	Breast cancer	20.47 (35)
	Cancer of unknown primary origin	11.70 (20)
	Cervical cancer	8.19 (14)
	Cholangiocarcinoma	7.60 (13)
	Colonic cancer	4.09 (7)
	Colorectal cancer	5.26 (9)
	Esophageal cancer	2.34 (4)
	Hodgkin’s lymphoma	4.68 (8)
	Lung cancer	5.85 (10)
	Nasopharyngeal cancer	1.17 (2)
	Ovarian cancer	9.94 (17)
	Prostate cancer	8.77 (15)
	Squamous cell carcinoma	2.92 (5)
	Total	100 (171)
Cancer stage	Stage I	12.87 (22)
	Stage II	19.30 (33)
	Stage III	28.07 (48)
	Stage IV	39.76 (68)
	Total	100 (171)
Pain severity	No pain	1.75 (3)
	Mild pain	22.81 (39)
	Moderate–severe pain	75.44 (129)
	Total	100 (171)

*n* indicates frequency.

*SD*, standard deviation.

### 3.2 Compliance rate to the ESMO pain management guidelines

The analysis of compliance with the ESMO pain management guidelines involved 171 patients admitted to the adult oncology ward. The pain severity and the treatment outcomes were regularly assessed using the Numeric Rating Scale (NRS) in 171 (100%) patients. Analgesics for chronic pain were prescribed regularly rather than on an “as needed” basis in 114 (66.70%) patients. The utilization of the WHO analgesic ladder was applied in 72 (42.10%) patients. In addition, 102 (59.65%) patients were informed about pain management and were encouraged to take an active role in their care. A laxative was routinely prescribed for both the prophylaxis and the management of opioid-induced constipation in 129 (75.44%) patients (**Table 3**). However, functional impairment due to moderate to severe pain was assessed in only 36 (21.05%) patients (**Table 3**). The subcutaneous (s.c.) route was considered as the first choice in 2 (1.16%) patients who were unable to receive opioids orally (**Table 3**).

**Table 3:** Frequency and percentage of compliance with the European Society of Medical Oncology (ESMO) guidelines in the management of adult oncologic pain at the University of Gondar Comprehensive and Specialized Hospital, 2024 (*N* = 171).

No.	Variable	Yes: *n* (%)	No: *n* (%)	Not applicable: *n* (%)
1	Were the pain severity and the treatment outcomes assessed regularly and consistently using the Visual Analogue Scale (VAS) or the Numeric Rating Scale (NRS)?	171 (100)	0	0
2	Was the onset of pain prevented by employing around-the-clock (ATC) administration, taking into account the half-life, bioavailability, and duration of action of different drugs?	108 (63.16)	60 (35.09)	3 (1.75)
3	Was functional impairment assessed for moderate to severe pain?	36 (21.05)	93 (54.39)	42 (24.56)
4	Were patients informed about pain and pain management and encouraged to take an active role in their pain management?	102 (59.65)	66 (38.6)	3 (1.75)
5	Were analgesics for chronic pain prescribed regularly and not on an “as required” basis?	114 (66.70)	45 (26.32)	12 (7.02)
6	Was the oral route of administration of analgesic drugs advocated as the first choice?	114 (66.67)	51 (29.82)	6 (3.51)
7	Was a combination of step 1 analgesics [e.g., paracetamol or non-steroidal anti-inflammatory drugs (NSAIDs)] for mild pain with step 2 (weak opioids) for moderate pain or step 3 analgesics (strong opioids) for severe pain utilized?	72 (42.10)	96 (56.14)	3 (1.75)
8	Was oral morphine the opioid of first choice for moderate to severe cancer pain?	39 (22.81)	90 (52.63)	42 (24.56)
9	Were different opioids considered in the absence of adequate analgesia (despite opioid dose escalation) or the presence of unacceptable opioid side effects?	51 (29.82)	51 (29.82)	69 (40.36)
10	Was the subcutaneous (s.c.) route considered as the first-choice route for patients unable to receive opioids by oral route?	2 (1.16)	4 (2.34)	165 (96.49)
11	Were laxatives routinely prescribed for both the prophylaxis and the management of opioid-induced constipation?	129 (75.44)	15 (8.77)	21 (15.79)
12	Were the side effects of opioid medications recorded?	69 (40.35)	75 (43.86)	21 (15.79)
13	Was cancer-related neuropathic pain treated using opioid combination therapies and carefully dosed adjuvants when opioids alone provided insufficient pain relief?	57 (33.33)	69 (40.35)	45 (26.32)
14	Were patients with neuropathic pain (NP) given either a tricyclic antidepressant (TCA) or an anticonvulsant?	51 (29.82)	66 (38.60)	54 (31.58

*n* indicates frequency.

**Figure 1:**
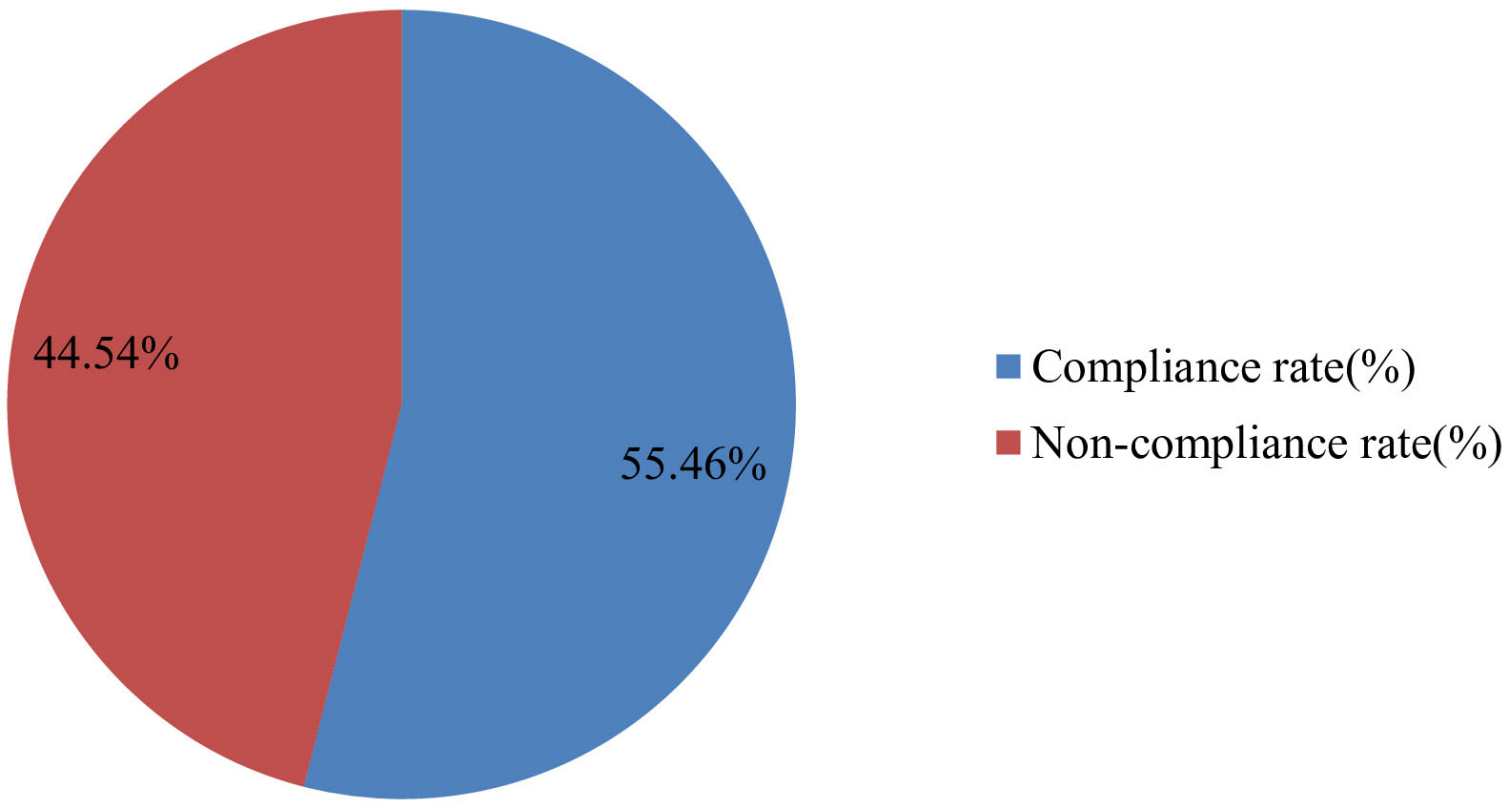
Overall compliance rate with the ESMO guideline in the management of adult oncologic pain at the University of Gondar Comprehensive and Specialized Hospital, 2024.

In this study, the overall compliance rate for oncologic pain management in accordance with the ESMO guidelines was 55.46% (**Figure 1**).

## 4 Discussion

This clinical audit evaluated the pain management practices for adult oncology patients based on the ESMO guidelines. In this study, the overall compliance rate with the ESMO guidelines for cancer pain management was 54%, which is comparable to the 56.6% compliance rate found in a study conducted in Nottingham City, England (16).

In the current study, the ages of the participants ranged from 23 to 85 years, with a mean age of 51.2 years. This is comparable to a clinical audit conducted at the University of Texas, USA, where the ages of participants also ranged from 23 to 85 years, with a mean age of 57 years (17).

In our study, the pain severity and the treatment outcomes were assessed regularly in 100% of the patients, which is almost in line with or comparable to the 89% in a study conducted in Australia. However, this result was higher than those in studies done in Nottingham City, the University of Texas (USA), and the University of Melbourne, which were 54%, 53%, and 51%, respectively (16, 18, 19). This variation could be due to the previous study being conducted earlier. During this time, there may have been improvements in the educational level of health professionals. Moreover, the difference in the sample size, which was higher in the current study, could have reduced errors, improved the accuracy, and provided a more reliable estimate of the true prevalence in the population.

In the current study, 59.65% of the patients were informed about pain and pain management and were encouraged to take an active role in their pain management. This finding is lower than that in a clinical audit conducted in Australia, where the result was 71% (18). This difference could be due to differences in the level of commitment of health professionals. This could also be due to differences in the training, workload, or attitude toward pain management education among healthcare providers, which can impact the amount of information provided to patients (20). In addition, time limitations, staffing shortages, and high patient loads can reduce opportunities for thorough discussions on pain management.

On the contrary, information dissemination in the current study was higher than that in the study conducted at the University of Texas (USA), which found that only 16% of outpatient charts and 19% of inpatient charts documented that patients were educated about their roles in managing pain, as well as the potential limitations and side effects of pain medications (17). This discrepancy could be due to differences in the study design. Unlike the current study, the previous study is retrospective, which could have resulted in missed or incomplete documentation. In addition, variations in the level of training and education among the health professionals involved in pain management could have also played a role.

In this study, utilization of the WHO analgesic leader or the combination of step 1 analgesics [e.g., paracetamol or non-steroidal anti-inflammatory drugs (NSAIDs)] for mild pain with step 2 analgesics (weak opioids) for moderate pain or step 3 analgesics (strong opioids) for severe pain was applied in 42.10% of the patients, which is close to the 43% in the study done in Nottingham City Hospital (16).

In the current study, analgesics for chronic pain were prescribed regularly and not on an “as required” basis in 66.70% of the patients, which is a lower than the 89% in the study conducted in Nottingham City Hospital, England, and the 94% in a study in Australia (16, 18). The rationale for this could be that, in the current study, the patients were already admitted to the ward and under the supervision of a healthcare professional. Their presence helped in the assessment of pain intensity, administering analgesics as necessary to minimize side effects and decreasing the use of addictive medications.

In this study, laxative was routinely prescribed for both the prophylaxis and the management of opioid-induced contraceptives in 75.44% of the patients, which is lower than that in the study performed in Nottingham City Hospital, England, which was 84% (16). This discrepancy may have stemmed from the current audit being conducted in a developing country, where there may be limited access to laxatives, differences in the clinical guidelines and laxative prescribing practices, variations in the awareness and training of the healthcare providers, and inconsistencies in patient assessment and monitoring.

### 4.1 Limitations of the audit

In this study, we focused on evaluating the adherence to the ESMO pain management guidelines at the University of Gondar Comprehensive Specialized Hospital. The study identified gaps in the adherence rates to these guidelines; however, the root causes of the observed issues were not determined.

## 5 Conclusion and recommendations

This audit assessed the compliance of adult oncologic pain management in the oncology ward of the University of Gondar Comprehensive Specialized Hospital using the ESMO guidelines. The results revealed that compliance with oncologic pain management indicators was below the optimal standard. It is crucial to prioritize the use of the s.c. route as the first choice for patients who are unable to take opioids orally, to adhere to the WHO pain management guidelines, to advocate for oral medication as the initial approach, and to assess the functional impairments of patients experiencing moderate to severe pain. Furthermore, we recommend the implementation of a regular training program on oncologic pain management for all healthcare professionals and the development of a standardized pain management protocol within the hospital.

## Data Availability

The original contributions presented in the study are included in the article/supplementary material. Further inquiries can be directed to the corresponding author.
